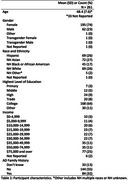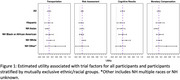# Assessing the Utility of Recruitment Strategies for Preclinical Alzheimer’s Disease Trials Among Racial and Ethnic Groups

**DOI:** 10.1002/alz70859_100590

**Published:** 2025-12-25

**Authors:** Mikaela K. Nishida, Megan G Witbracht, Michelle M Nuño, Crystal M Glover, Jason Karlawish, Kristin Harkins, Daniel L Gillen, Joshua D Grill

**Affiliations:** ^1^ University of California, Irvine, Irvine, CA USA; ^2^ Institute for Memory Impairments and Neurological Disorders, University of California, Irvine, Irvine, CA USA; ^3^ University of Southern California, Los Angeles, CA USA; ^4^ The UC Irvine Institute for Memory Impairments and Neurological Disorders, Irvine, CA USA; ^5^ University of Pennsylvania Perelman School of Medicine, Philadelphia, PA USA

## Abstract

**Background:**

Trialists face many challenges in recruiting participants to preclinical Alzheimer’s disease (AD) clinical trials. The utility of strategies such as reducing logistical barriers and offering of incentives has been analyzed, but few studies have examined potential differences in utility across racial and ethnic groups.

**Methods:**

We conducted a conjoint experiment to assess the utility of four factors – providing transportation, providing lifetime dementia risk assessment, providing cognitive test summaries, and financial compensation – on participants’ willingness to participate in a preclinical trial. Participants rated each of 16 hypothetical preclinical AD trial vignettes on a 7‐point Likert scale ranging from “Definitely would not participate” to “Definitely would participate.” We analyzed the utility of each of the factors using generalized estimating equations. We repeated the same analysis for 5 mutually exclusive ethnic and racial groups (i.e. Hispanic, non‐Hispanic (NH) Asian, NH Black or African American, NH White, NH Other) based on participant self‐reported data.

**Results:**

Out of N=261 participants, 69 participants self‐identified as Hispanic, 72 as NH Asian, 45 as NH Black or African American, 69 as NH White, 5 as NH Other (i.e. multiple races or not reported), and 1 did not report. We estimated that the overall utility was approximately 0.25 points (95% CI: 0.17, 0.33; P < 0.001) for providing transportation, 0.10 points (95% CI: 0.06, 0.15; P <0.001) for providing a lifetime risk assessment for developing AD dementia, 0.30 points (95% CI; 0.21, 0.39; P<0.001) for providing a summary of the cognitive test results, and 0.30 points (95% CI: 0.22, 0.37; P<0.001) for providing higher monetary compensation. Figure 1 displays differences in utility between ethnic and racial groups. Compared to the full cohort, transportation and higher monetary compensation were associated with less utility in NH Asian participants, and dementia risk estimates and cognitive test results were associated with higher utility in NH White participants.

**Conclusions:**

Strategies such as providing transportation, risk estimates for developing AD dementia, cognitive test results, and higher monetary compensation all were associated with greater willingness to participate in AD clinical trials overall, but demonstrated few differences in magnitude among ethnic and racial groups.